# Noncoding RNA, antigenic variation, and the virulence genes of *Plasmodium falciparum*

**DOI:** 10.1186/1741-7007-9-50

**Published:** 2011-07-19

**Authors:** A Taylor Bright, Elizabeth A Winzeler

**Affiliations:** 1 Biomedical Sciences Program, University of California, San Diego, 9500 Gillman Dr., La Jolla, CA 92093, USA; 2 Department of Genetics, The Scripps Research Institute, 10550 North Torrey Pines Rd, La Jolla, CA 92037, USA; 3 Department of Cell Biology, Genomics Institute of the Novartis Research Foundation, 10675 John Jay Hopkins Drive, San Diego, CA 92121, USA

## Abstract

Long non-coding RNAs (lncRNA) are being increasingly recognized as important regulators of gene expression. A recent paper in *Genome Biology *reports the identification of a lncRNA family in *Plasmodium falciparum*, the cause of the most deadly form of malaria, that may help to explain the mechanism of antigenic variation in virulence genes of this important pathogen.

See Research article: http://genomebiology.com/2011/12/6/R56/abstract

## Commentary

Globally there are 300 to 450 million cases of malaria each year, with the most severe form of human malaria being caused by the apicomplexan parasite *Plasmodium falciparum *[1].

**Figure 1:**
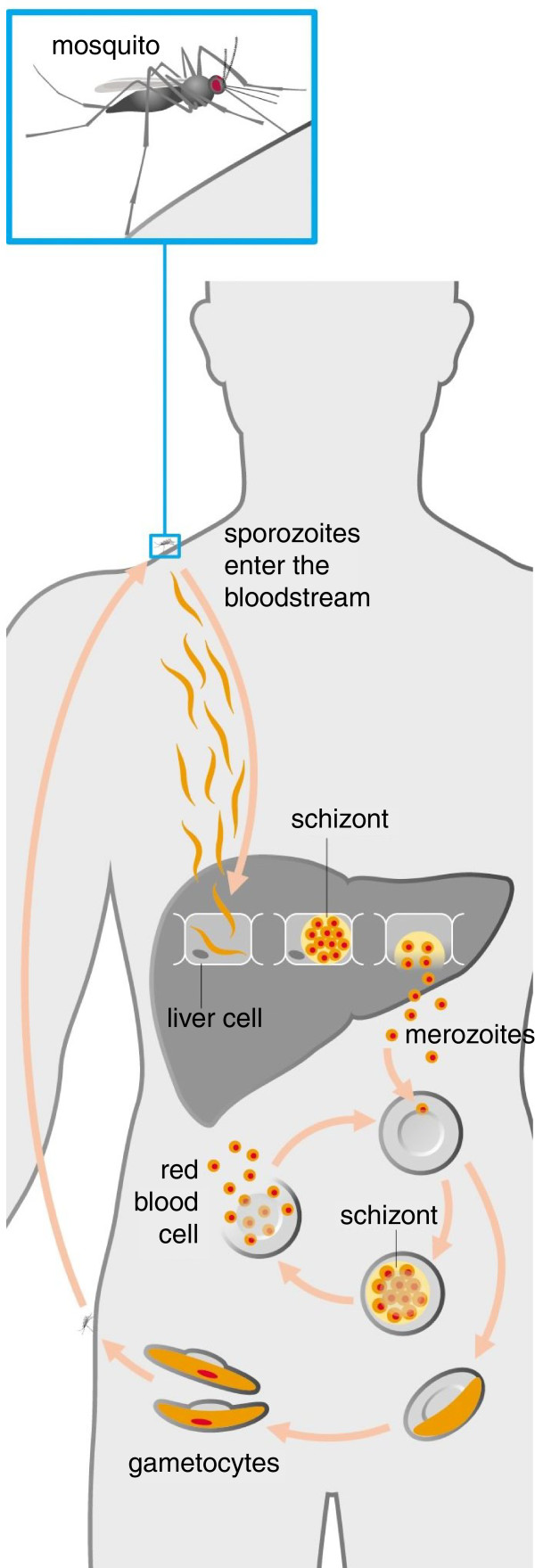
**Lifecycle of the malaria parasite**. After a human host is infected by the mosquito vector immature malaria parasites known as sporozites migrate to the liver where they invade hepatocytes and mature into schizonts, which then rapidly multiply into merozoites. After a 10 to 14 day incubation period, the hepatocytes rupture and thousands of merozoites emerge. Merozoites then invade erythrocytes where they extensively remodel their host cell, including exporting PfEMP1 to the red blood cell surface. Merozoites can continue in the asexual cycle or may develop into sexual stage gametocytes capable of infecting a mosquito and beginning the cycle anew. (Reproduced with permission from Figure 14-8 in DeFranco AL, Locksley RM, Robertson M: Immunity: The Immune Response in Infectious and Inflammatory Disease. Oxford: Oxford University Press; 2007.).

A critical determinant of virulence in this pathogen is a family of adhesion molecules, the *P. falciparum *erythrocyte membrane protein 1s (PfEMP1s). At any one time, one member of this family is expressed in a mutually exclusive manner and exported to the surface of infected red blood cells, causing them to bind to endothelial cells and leading to sequestering of the infected red blood cells in the microvasculature. Sequestration prevents the infected red blood cell from being removed from the circulation in the spleen, thus prolonging the infection and allowing the parasite more time to develop gametocytes, which are sexual stage forms capable of infecting the mosquito vector (Figure 1). Sequestration also allows infected red blood cells to accumulate in the microvasculature, causing occlusion of blood vessels that can lead to vasculature rupture and hemorrhage [2].

PfEMP1s, which along with being a major virulence determinant are also a major antigenic target of the host immune response, are encoded by the *var *genes, which comprise a highly variable family of 60 genes, located in six internal gene clusters and at the ends of the parasite's chromosomes in the subtelomeric regions. Only one of these genes is expressed by each individual parasite at a time [3], but switching between the different genes occurs, defeating the adaptive immune response of the human host. The mechanism by which the parasite transcriptionally silences all but one of the 60 or so *var *genes is poorly understood, but epigenetic mechanisms are implicated [4]. The discovery by Broadbent *et al. *[5] of a family of 22 long non-coding RNAs (lncRNAs) that map to the chromosome ends, where they are adjacent to *var *genes, suggests, for the first time, mechanisms that might underlie the epigenetic regulation of *var *genes.

## Regulation of virulence

Epigenetic changes underlying gene expression are generally mediated by modifications to histones that control chromatin remodeling; and although *P. falciparum *lacks the diversity of transcription factors characteristic of other eukaryotic organisms, it is known to have a full repertoire of histone modifying genes [6].

The epigenetic changes governing *var *gene expression and switching are well studied. Activation of *var *genes is marked by histone 3 lysine 9 (H3K9) acetylation and H3K4me2/me3 [4], while *var *gene repression is marked by enrichment of the canonical repressive epigenetic marker H3K9me3, which is bound by *P. falciparum *heterochromatin protein 1 (PfHP1), thereby nucleating heterochromatin formation [7]. H3K9me3 and PfHP1 binding is enriched throughout not just the *var *gene families but also the neighboring telomere associated repeat element (TARE) regions on all chromosomes [8]. The TARE regions and a subtelomeric class of *var *genes - known as the upsB-type *var *genes - are also enriched in the *cis*-acting element SPE2, a bipartite 12 base pair sequence critical for regulation of *var *gene silencing that has recently been shown to be bound by a member of the ApiAP2 transcription factor family, *P. falciparum *SPE2 interacting protein (PfSIP2) [9].

But while the epigenetic markers that delineate active and silenced *var *genes in *P. falciparum *are well understood, and a few key proteins and DNA elements have been identified, virtually nothing is known about the mechanisms by which a single *var *gene is exclusively activated. The lncRNA family discovered by Broadbent *et al. *- termed the lncRNA-TAREs because they exclusively map to the TARE regions on the ends of the chromosomes - is implicated in the mechanism of *var *gene regulation by two observations. First, the lncRNA-TAREs contain the majority of the SPE2 binding sites. The only other clusters of SPE2 sites are in the promoters of upsB-type *var *genes [10]. And second, the lncRNA-TAREs are found adjacent to all of the upsB-type *var *genes. Moreover, induction of lncRNA-TAREs occurs directly after DNA transcription, when epigenetic memory marks would be expected to be initiated in new chromatin.

Earlier work on non-protein coding RNAs had shown the presence of ncRNAs from the telomeric and subtelomeric regions [11], but there has as yet been only a vague hypothesis that these ncRNAs play a role in telomere stability and/or are involved in regulation of *var *genes. In their recent work, Broadbent *et al. *definitively link a family of lncRNAs to *var *genes for the first time by discovering that the subtelomeric SPE2 clusters are transcribed into non-coding RNAs. Taken together, the position within the genome and the transcriptional profile of the lncRNA-TAREs and the presence of *var *gene-associated motifs within the lncRNA-TARE sequences suggest that this novel non-protein coding RNA family may play a part in regulation of *var *genes, possibly through chromatin remodeling.

## lncRNA-TAREs as a potential *var *gene regulator

Building on what is known about the function of lncRNAs in other organisms, Broadbent *et al. *propose possible mechanisms by which lncRNA-TAREs might impact gene expression. These proposed mechanisms involve regulating *trans*-acting proteins and/or recruiting these factors or chromatin-modifying complexes to their sites of action. Lending credence to these proposed mechanisms, recent evidence has demonstrated that lncRNAs function in a similar role in *var*-like variegated gene expression in humans. Two examples from the Homeobox (HOX) gene clusters in humans demonstrate the diversity of functions carried out by lncRNAs with regards to gene regulation. First, HOTAIR, a 2.2 kb lncRNA in the HOXC locus, is known to interact with Polycomb repressive complex 2 (PRC2) to silence, in *trans*, the HOXD locus [12]. And second, another lncRNA, termed HOTTIP, has recently been identified that promotes activation of genes in the 5' region of the HOXA locus in *cis *by binding to *trans*-acting factors directly and targeting them to the HOXA complex, thereby enriching the area in epigenetic activation markers [13].

At this stage, it is a matter of speculation how the newly discovered lncRNA-TAREs might account for the specialized regulation of the *var *genes of *P. falciparum*; but they add an important element to the possible mechanism whereby this important human pathogen evades elimination by the immune system.
